# Supplementary figures and images for: HSP90α Mediates Sorafenib Resistance in Human Hepatocellular Carcinoma by Necroptosis Inhibition under Hypoxia
Source: Cancers (Basel). 2021 Jan 11;13(2):243. doi: 10.3390/cancers13020243 (PMC7827218; doi:10.3390/cancers13020243)

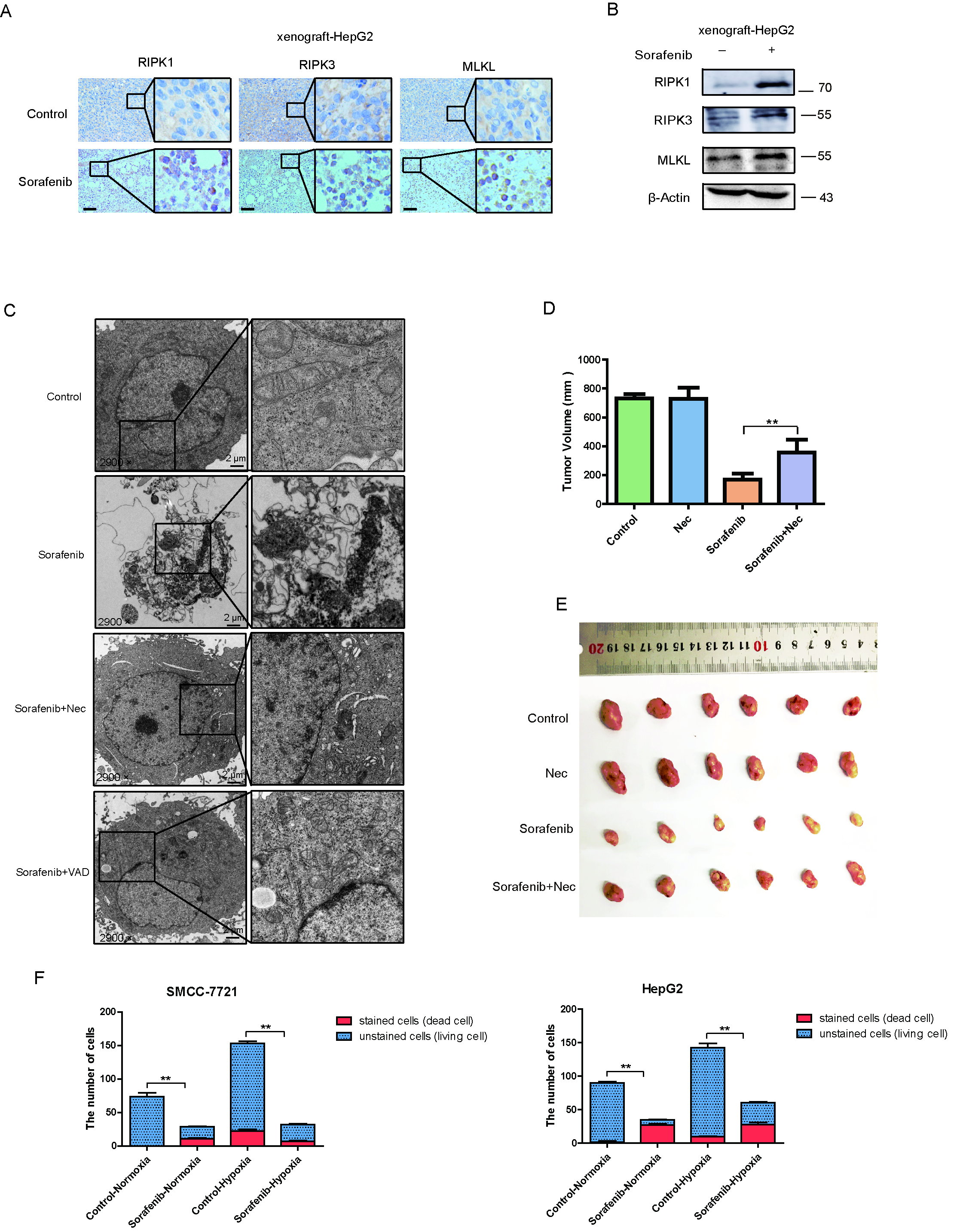

Supplement: Supplementary file 1 [file cancers-13-00243-s001.zip › Figure S1.tif]

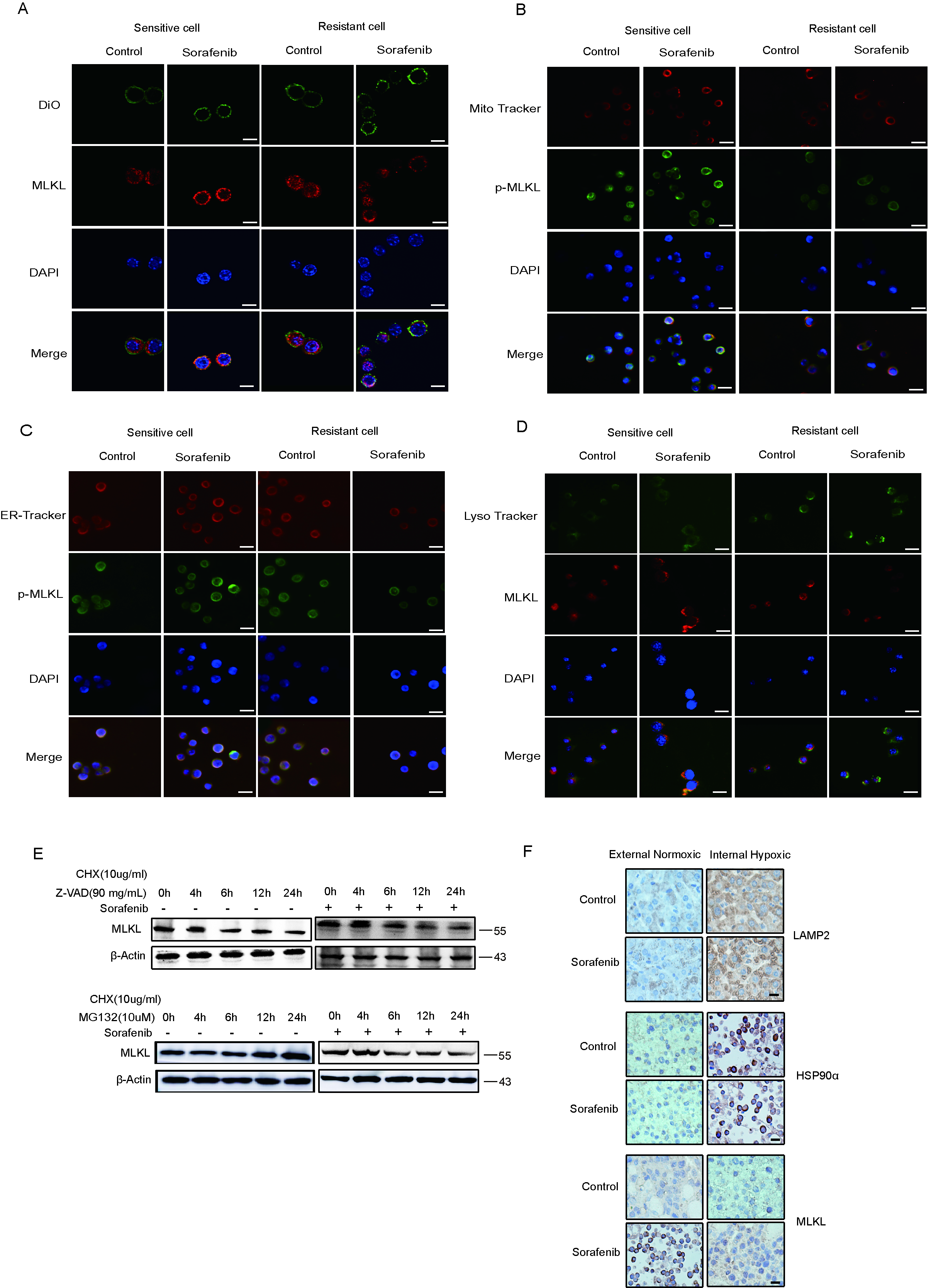

Supplement: Supplementary file 1 [file cancers-13-00243-s001.zip › Figure S2.tif]

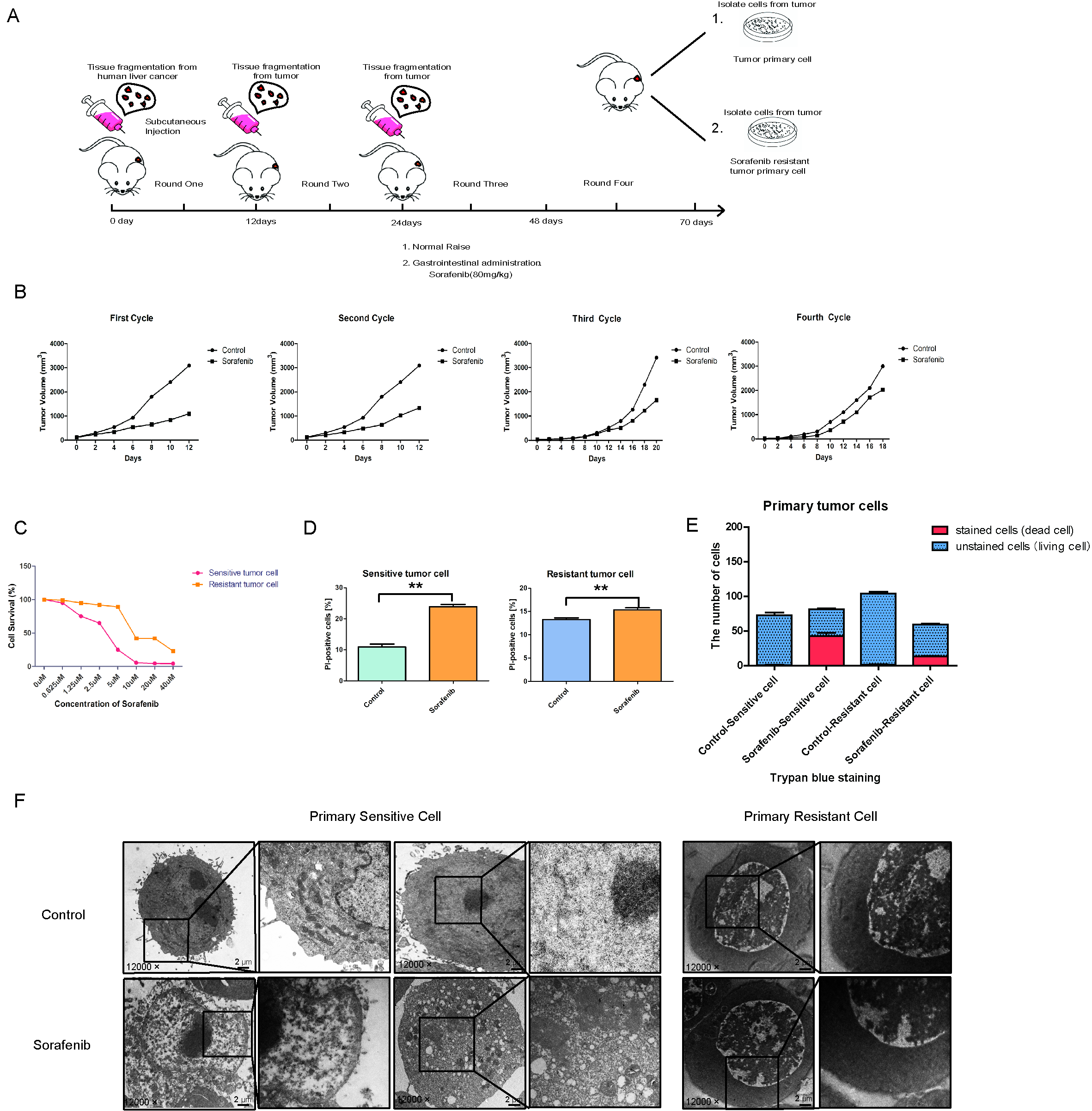

Supplement: Supplementary file 1 [file cancers-13-00243-s001.zip › Figure S3.tif]

Figure 1

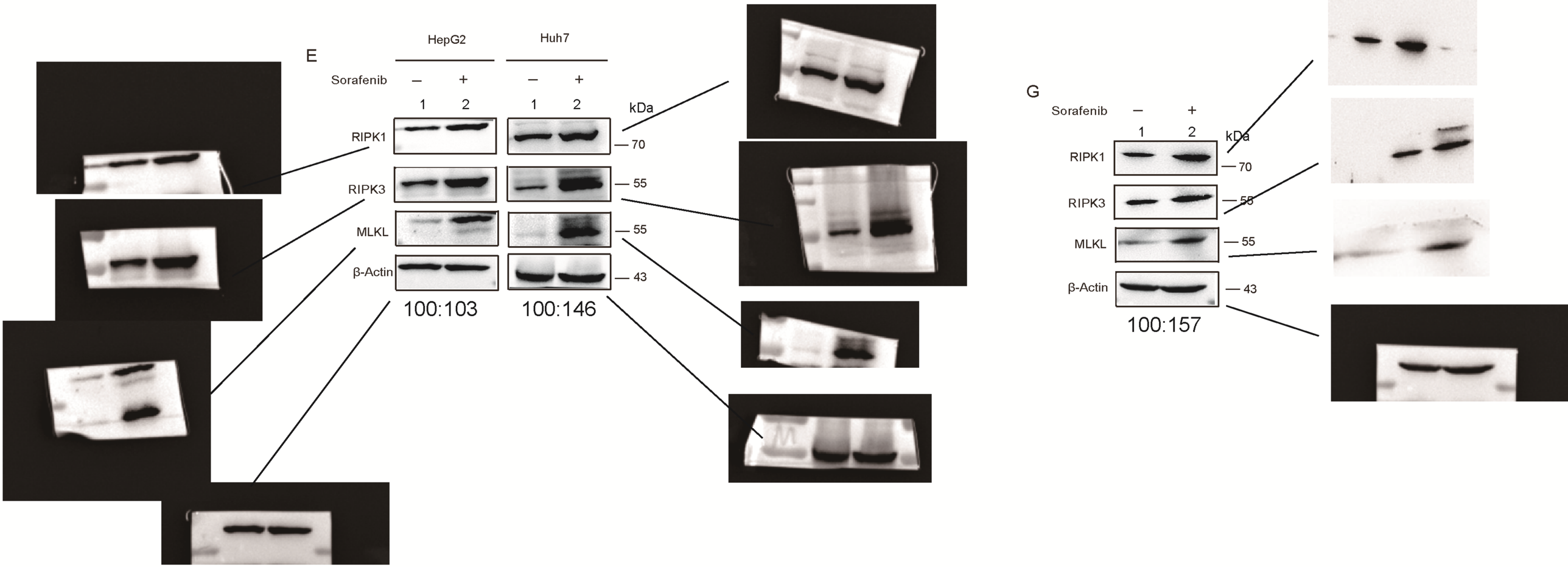

Figure 2

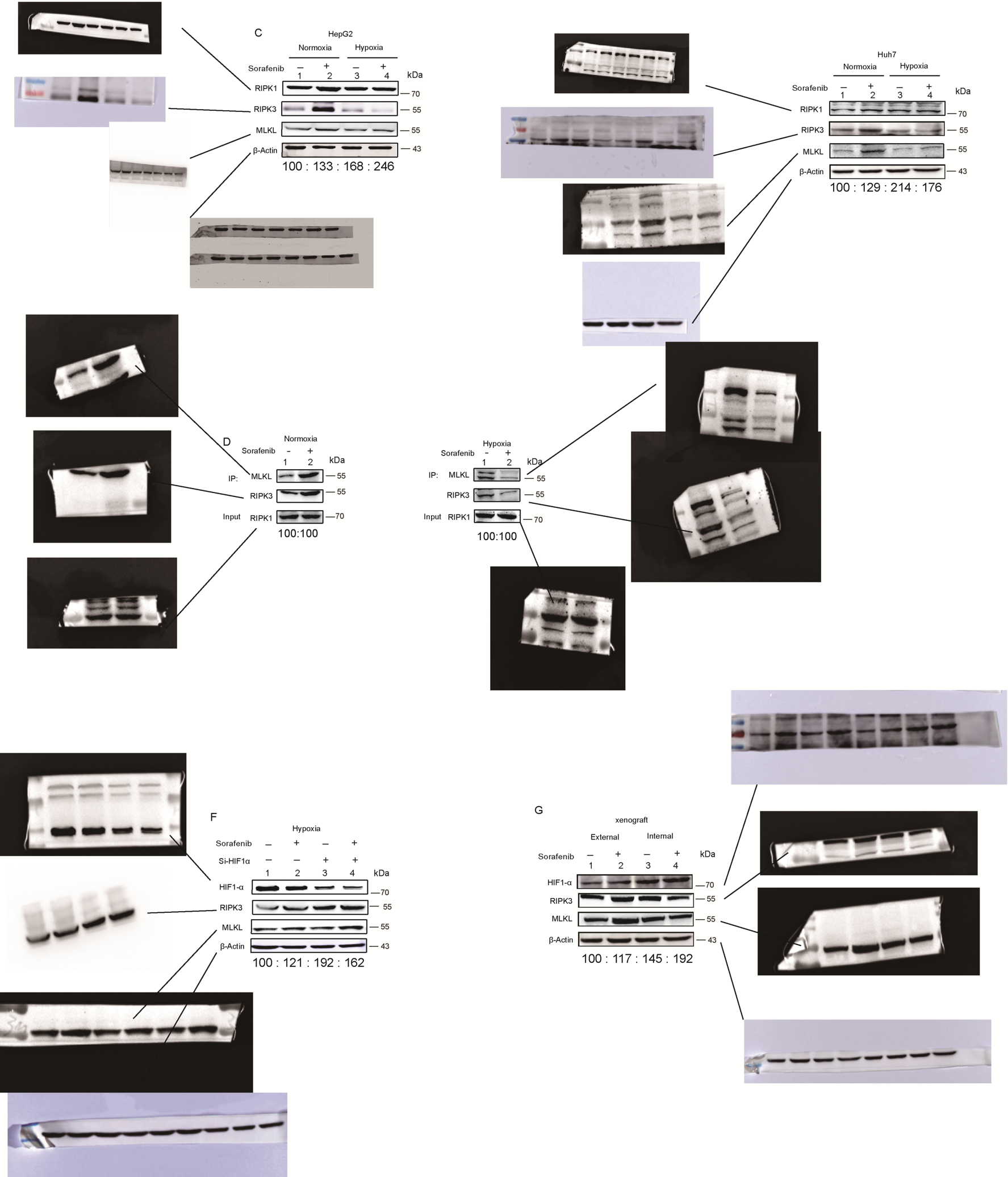

Supplement: Supplementary file 1 [file cancers-13-00243-s001.zip › Figure S4.pdf]
