# Supplementary material for: HSP90α Mediates Sorafenib Resistance in Human Hepatocellular Carcinoma by Necroptosis Inhibition under Hypoxia
Source: Cancers (Basel). 2021 Jan 11;13(2):243. doi: 10.3390/cancers13020243 (PMC7827218; doi:10.3390/cancers13020243)

Figure 3

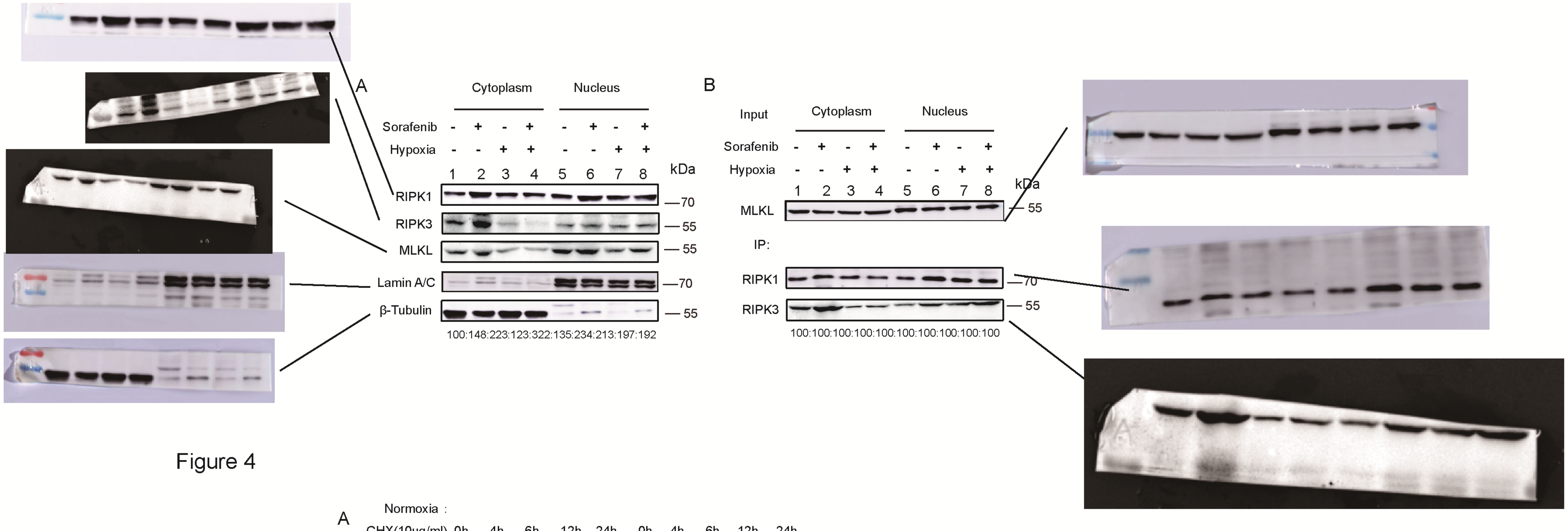

Figure 4

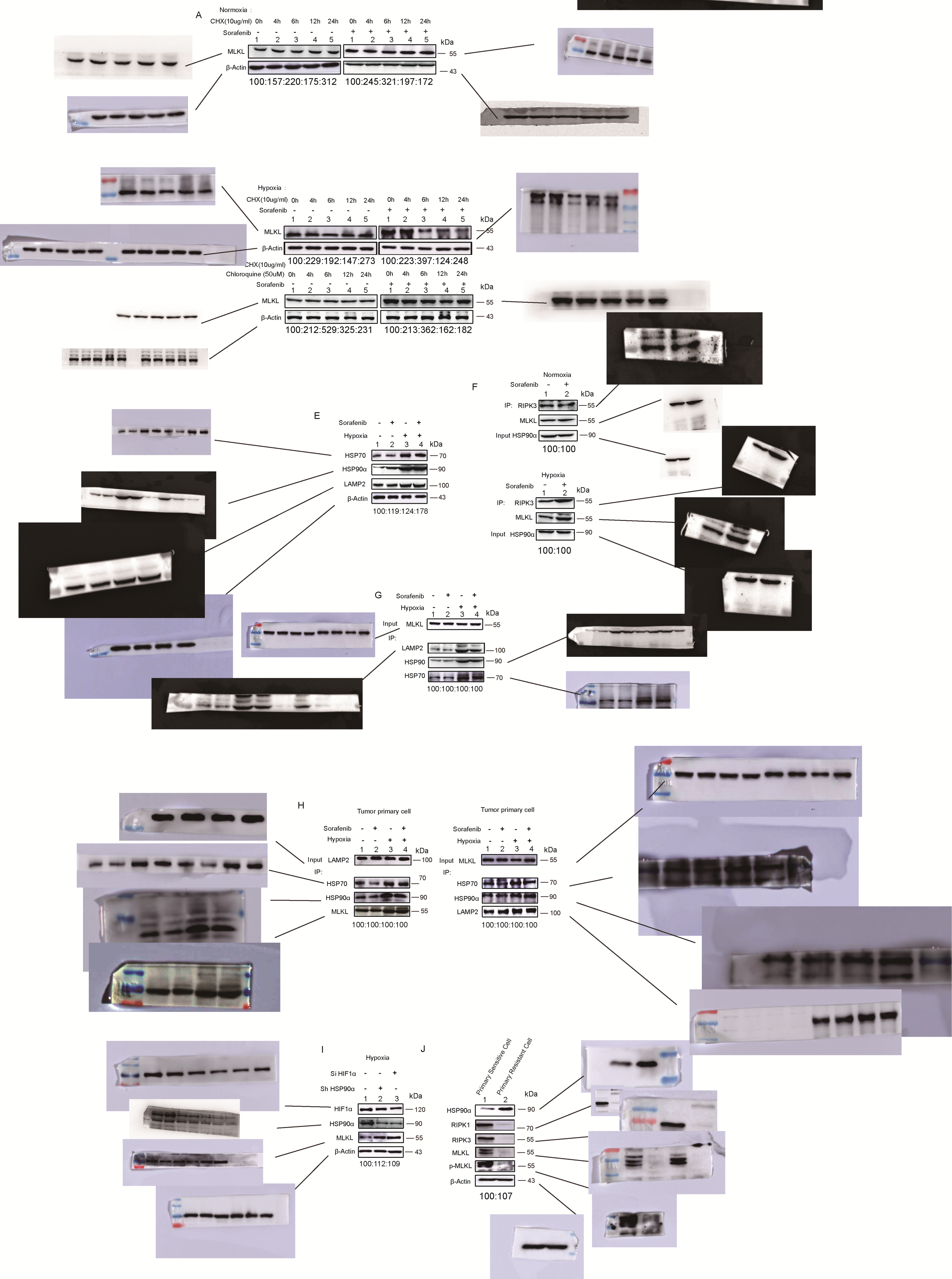

Supplement: Supplementary file 1 [file Figure S5.pdf › Figure S5.pdf]
