# Supplementary material for: HSP90α Mediates Sorafenib Resistance in Human Hepatocellular Carcinoma by Necroptosis Inhibition under Hypoxia
Source: Cancers (Basel). 2021 Jan 11;13(2):243. doi: 10.3390/cancers13020243 (PMC7827218; doi:10.3390/cancers13020243)

Figure 5

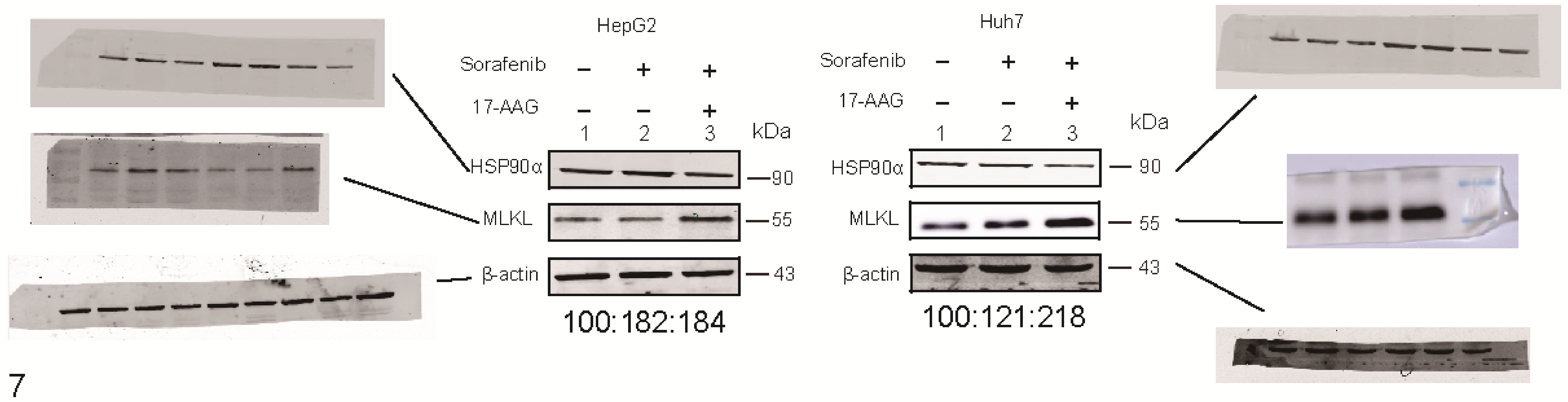

Figure 7

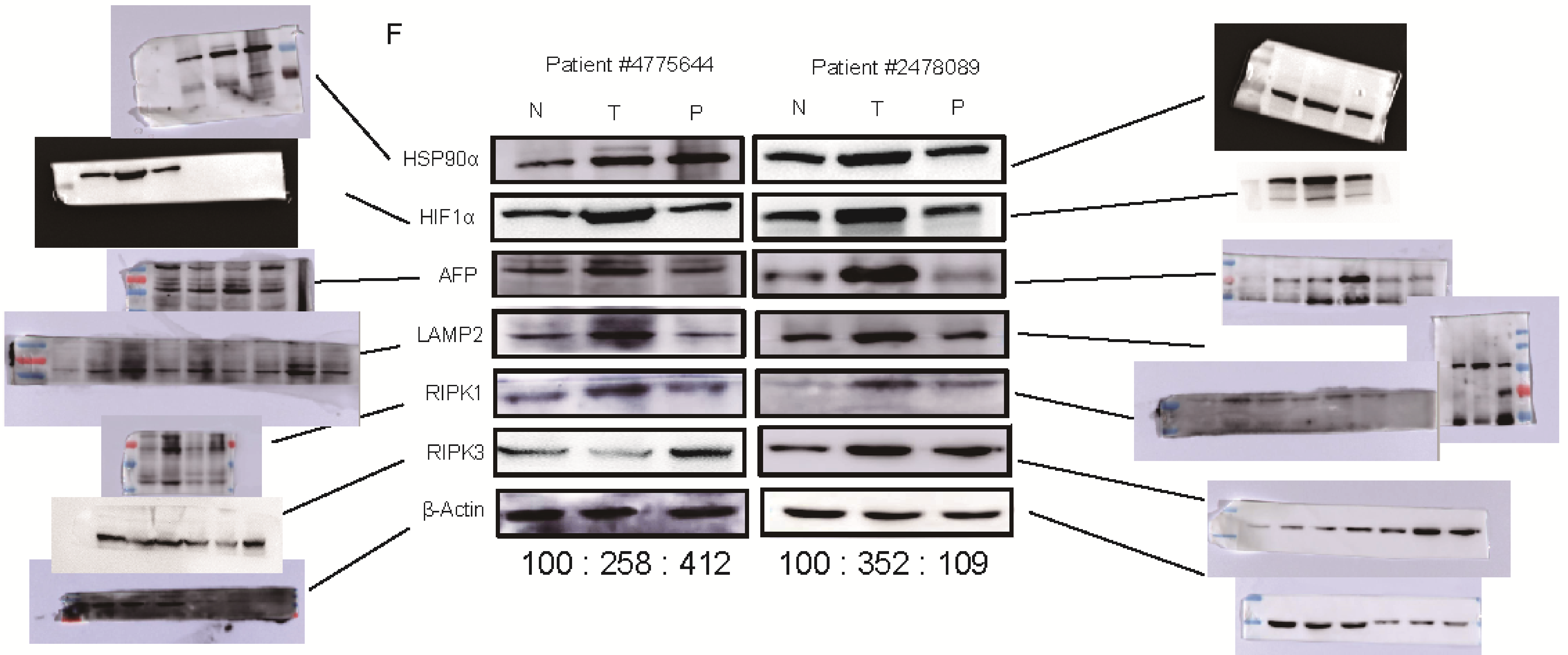

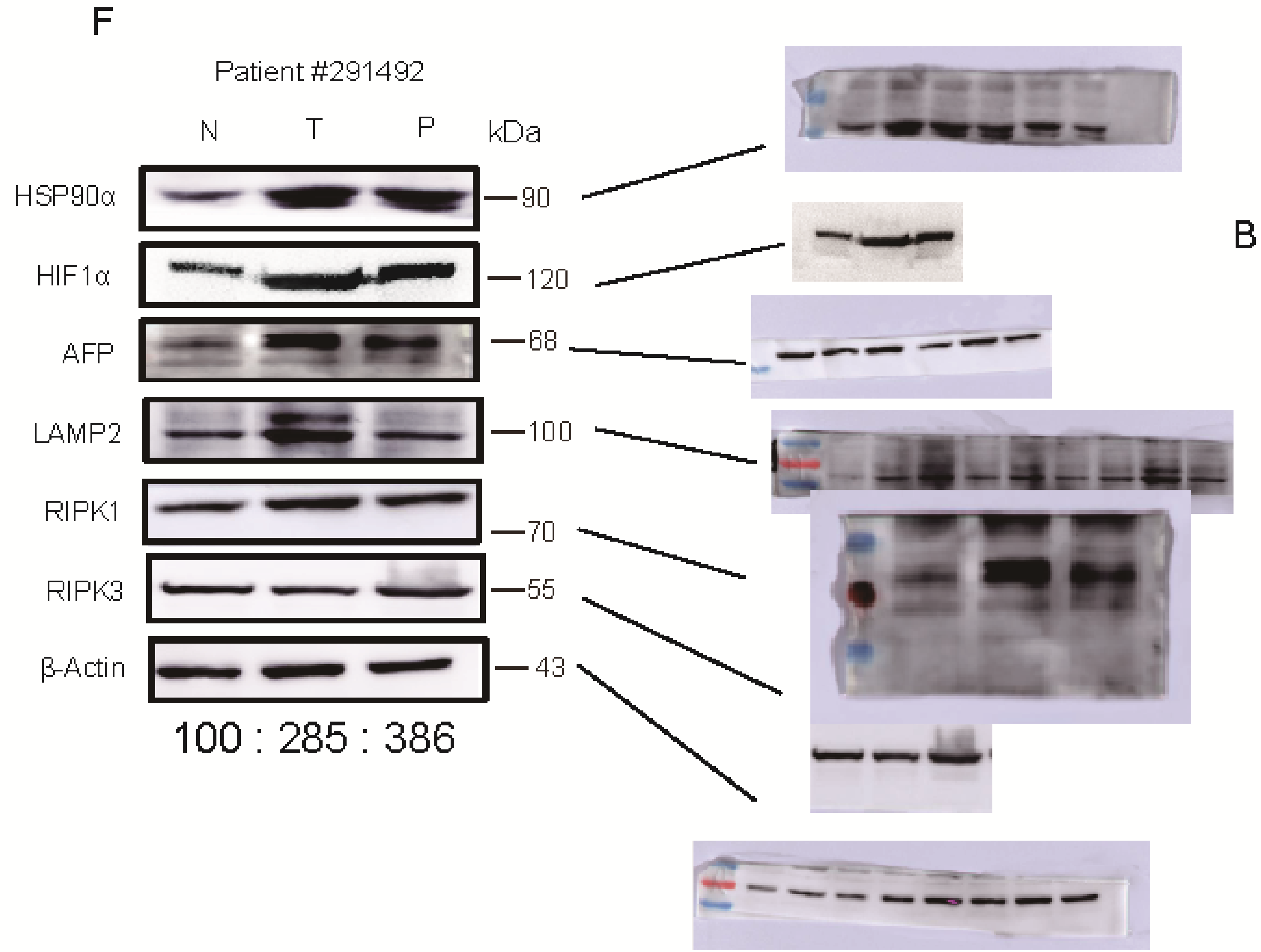

Supply 2

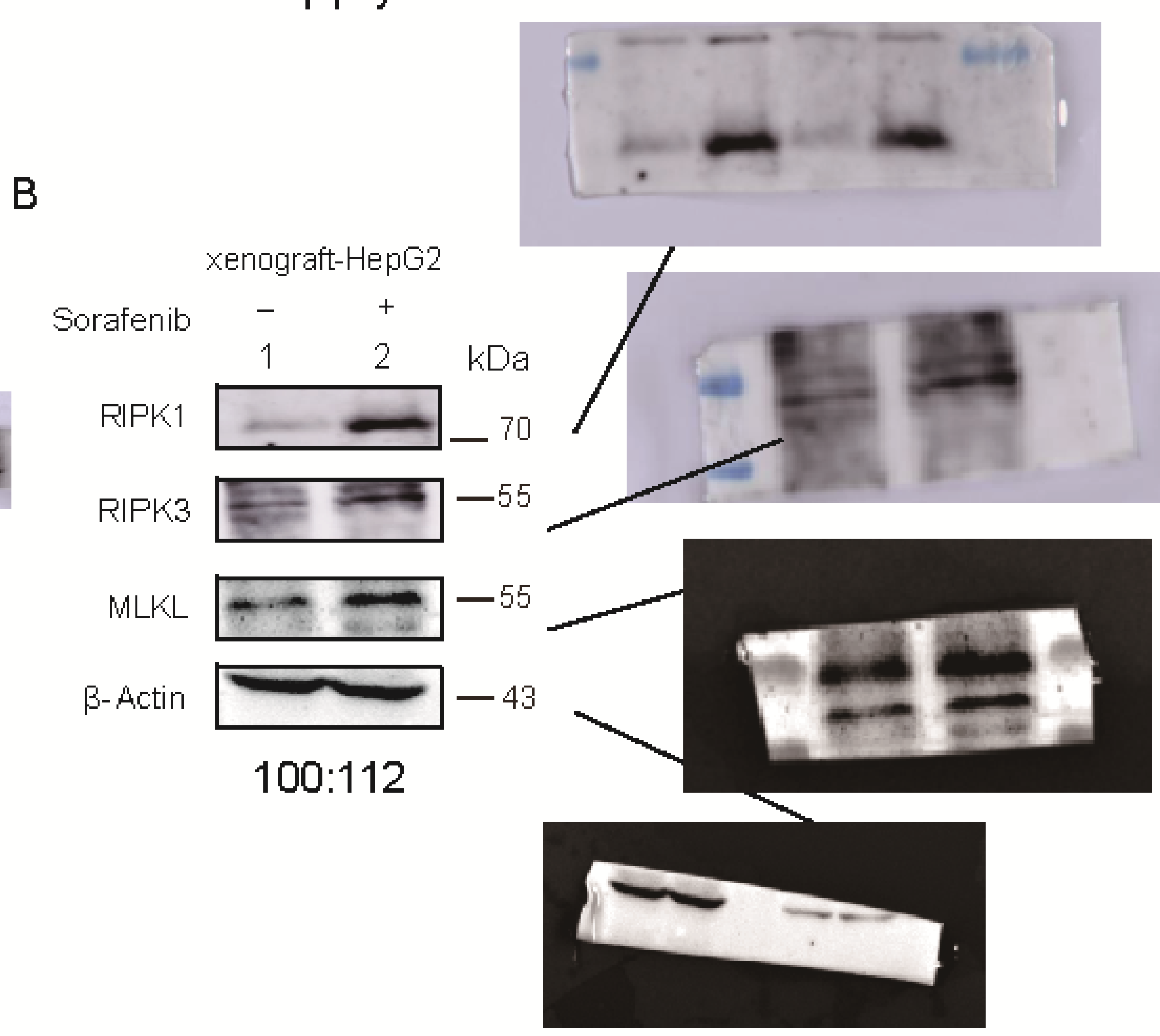

Supply 3

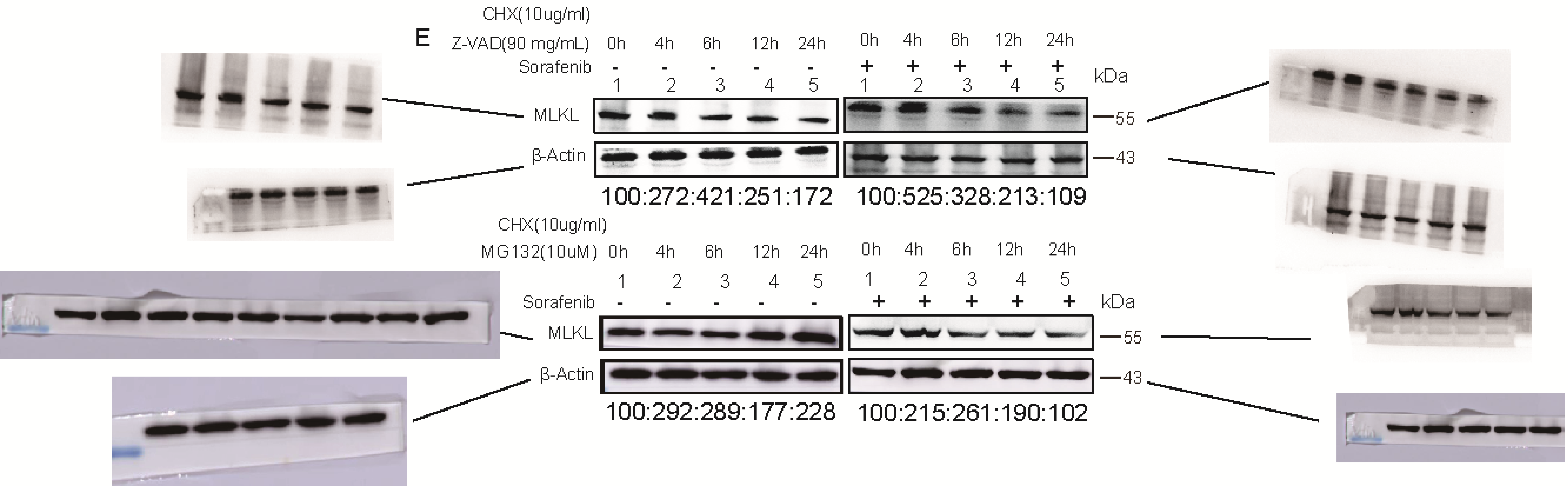

Supplement: Supplementary file 1 [file Figure S6.pdf › Figure S6.pdf]
